# Mice with RyR1 mutation (Y524S) undergo hypermetabolic response to simvastatin

**DOI:** 10.1186/2044-5040-3-22

**Published:** 2013-09-03

**Authors:** Mark Knoblauch, Adan Dagnino-Acosta, Susan L Hamilton

**Affiliations:** 1 Department of Molecular Biology and Biophysics, Baylor College of Medicine, 1 Baylor Plaza, Houston, TX 77030, USA

**Keywords:** Statin-induced myopathy, Simvastatin, RyR1, Myopathy, Calcium signaling

## Abstract

**Background:**

Statins are widely used drugs for the treatment of hyperlipidemia. Though relatively safe, some individuals taking statins experience rhabdymyolysis, muscle pain, and cramping, a condition termed statin-induced myopathy (SIM). To determine if mutations in the skeletal muscle calcium (Ca^2+^) release channel, ryanodine receptor type 1 (RyR1), enhance the sensitivity to SIM we tested the effects of simvastatin, the statin that produces the highest incidence of SIM in humans, in mice with a mutation (Y524S, ‘YS’) in RyR1. This mutation is associated with malignant hyperthermia in humans. Exposure of mice with the YS mutation to mild elevations in environmental temperature produces a life-threatening hypermetabolic response (HMR) that is characterized by increased oxygen consumption (VO_2_), sustained muscle contractures, rhabdymyolysis, and elevated core body temperature.

**Methods:**

We assessed the ability of simvastatin to induce a hypermetabolic response in the YS mice using indirect calorimetry and to alter Ca^2+^ release via RyR1 in isolated flexor digitorum brevis (FDB) fibers from WT and YS mice using fluorescent Ca^2+^ indicators. We also tested the ability of 5-aminoimidazole-4-carboxamide ribonucleoside (AICAR) to protect against the simvastatin effects.

**Results:**

An acute dose of simvastatin triggers a hypermetabolic response in YS mice. In isolated YS muscle fibers, simvastatin triggers an increase in cytosolic Ca^2+^ levels by increasing Ca^2+^ leak from the sarcoplasmic reticulum (SR). With higher simvastatin doses, a similar cytosolic Ca^2+^ increase occurs in wild type (WT) muscle fibers. Pre-treatment of YS and WT mice with AICAR prevents the response to simvastatin.

**Conclusions:**

A mutation in RyR1 associated with malignant hyperthermia increases susceptibility to an adverse response to simvastatin due to enhanced Ca^2+^ release from the sarcoplasmic reticulum, suggesting that RyR1 mutations may underlie enhanced susceptibility to statin-induced myopathies. Our data suggest that AICAR may be useful for treating statin myopathies.

## Background

Statins (3-hydroxy-3-methylglutaryl coenzyme-A (HMG-CoA) reductase inhibitors) are cholesterol-lowering drugs that have proven effective in decreasing low-density lipoprotein (LDL) levels and improving overall health [1]. For the majority of patients, statins are well tolerated with few side effects. However, up to 10% of patients on a statin regimen display muscle-related symptoms including soreness, fatigue, and an increase in circulating levels of muscle-specific proteins (for example creatine kinase (CK)) that results in a condition termed statin-induced myopathy (SIM) [2,3]. A mechanism to explain the underlying cause of SIM has yet to be elucidated.

One emerging theory of SIM has centered on statins’ potential to modulate intramyofiber calcium (Ca^2+^) homeostasis [4-6]. This theory stems in part from the finding that the direct application of simvastatin to healthy human myofibers triggers a significant increase in cytosolic Ca^2+^[7]. The sudden release of Ca^2+^ in response to direct application of statins *in vitro* has been suggested to originate from both mitochondria and the sarcoplasmic reticulum (SR) [5,8,9], the predominant Ca^2+^ storage organelle within the myofiber. The potential involvement of the SR in statin-induced Ca^2+^ release is particularly intriguing given the recent findings that mutations in ryanodine receptor type 1 (RyR1), the Ca^2+^ release channel of the SR, may underlie some instances of SIM [10,11]. Mutations in RyR1 are known to produce malignant hyperthermia (MH), a life-threatening condition where uncontrolled release of Ca^2+^ within the myofiber is triggered by exposure to certain volatile inhalants, elevated temperature, or exercise [12,13]. This uncontrolled release of Ca^2+^ results in sustained muscle contractions, elevated core temperature, rhabdomyolysis and, if unabated, death [12].

At present our understanding of the link between RyR1 mutations and statin myopathies has been limited to *in vitro* work with muscle biopsies. Metterlein *et al.* found that biopsied muscle from MH-sensitive swine exhibit contraction upon exposure to statins *in vitro*[10]. Similarly, Guis *et al.* found that muscle biopsies from seven of nine human subjects exhibiting the signs of SIM expressed abnormal *in vitro* contracture tests (IVCT) used to screen for susceptibility to MH [11].

These *in vitro* findings combined with evidence that simvastatin modifies Ca^2+^ homeostasis suggest that RyR1 mutations may underlie enhanced susceptibility to SIM. We developed a mouse model (Y524S, ‘YS’) with a RyR1 knock-in mutation of tyrosine 524 to serine [13], which in humans (Y522S) is associated with MH [13]. Mice homozygous for the mutation die at birth, while heterozygous YS mice exhibit a hypermetabolic response (HMR) to elevated (37°C) temperature, volatile anesthetics, or exercise in a warm environment. These mice are a valuable tool for studying some RyR1-associated disorders. The purpose of the present study was to determine whether mice with this RyR1 mutation (Y524S) display HMR when given simvastatin and to evaluate the effects of simvastatin on intramyofiber Ca^2+^ homeostasis.

## Methods

### Animal care and handling

All procedures were approved by the Institutional Animal Care and Use Committee at Baylor College of Medicine, Houston, TX, USA. As previously described, male RyR1^Y524S/WT^ (‘YS’) mice were developed and used in conjunction with wild type (WT) littermate controls at 8 to 10 weeks of age. Mice were maintained on a 12:12 light:dark cycle, had *ad libitum* access to water and standard mouse chow, and were limited to normal cage activity only. All mice were sacrificed at the same time of day, consisting of cervical dislocation after anesthetization under isoflurane.

### Statin preparation

Simvastatin was purchased from the manufacturer (LKT Laboratories, St Paul, MN, USA) in powder form. For studies involving injection into mice for indirect calorimetry, simvastatin powder was dissolved in dimethyl sulfoxide (DMSO). For single-fiber perfusion work, a 12 mM simvastatin stock was prepared in 10% EtOH similar to previous studies [14]. After adjusting the pH to 7.0, the solution was brought up to 12 mM concentration in Tyrode’s solution containing 121 mM NaCl, 5 mM KCl, 1.8 mM CaCl_2_, 500 μM MgCl_2_, 400 μM NaH_2_PO_4_, 100 μM EDTA, 5.5 mM glucose, and 24 mM NaHCO_3_. Separately, a vehicle-only stock was prepared identically but without the addition of simvastatin. These prepared stocks were aliquoted and frozen at −80°C until use.

### Indirect calorimetry monitoring of VO_2_ max

Those YS and WT mice used to determine the effects of statin dosing *in vivo* were removed from their cage, weighed, and injected IP with an 30-80 mg/kg dose of either simvastatin dissolved in DMSO or DMSO alone (‘vehicle’). The mice were then returned to their cages for 30 minutes, after which they were placed individually into an environmental chamber at 32°C containing indirect calorimetry chambers (Oxymax System, Columbus Instruments, Columbus, OH, USA), which allowed for monitoring of maximum oxygen consumption (VO_2_ max (mL/kg/min)). Separately, to evaluate the effectiveness of a pharmaceutical agent shown previously to prevent heat-induced HMR response in YS mice [15], additional YS mice were injected IP with a 600 mg/kg dose of 5-aminoimadazole-4-carboxamide ribonucleoside (AICAR) 20 minutes after simvastatin injection.

### Fiber isolation

For mice destined for single-fiber Ca^2+^ study, the flexor digitorum brevis (FDB) muscle was removed and immediately placed into Dulbecco’s modified Eagle’s medium (DMEM) containing 3 mg/mL collagenase and 10% (v/v) fetal bovine serum. After a 2-hour incubation at 37°C, whole FDB muscles were transferred to 1 mL of DMEM and plunged ten times through a 1 mL pipette tip to separate individual fibers. Next, 150 μL of DMEM containing separated FDB fibers was placed onto a 25 mm glass coverslip that had been incubated for 2 hours with 20 μg/mg of laminin in PBS and then subjected to two washes in PBS and a final wash in DMEM. Prior to use, plated fibers were incubated overnight at 37°C in DMEM containing antibiotic-antimycotic (Gibco, Carlsbad, CA, USA).

### Isolated fiber preparation and imaging

To assess the sensitivity to simvastatin, after the overnight incubation the fibers were next incubated for 1 hour at room temperature in either DMEM containing (10 μM) Fura-2 acetoxymethyl ester (Fura-2 AM) or 30 minutes in DMEM containing (5 μM) Mag-fluo-4, with (20 μM) contraction-inhibitor 4-methyl-N-(phenylmethyl)benzenesulfonamide (BTS). Fibers were placed in a temperature controlled chamber (Dagan Corporation, Minneapolis, MN, USA) on the stage of an inverted epifluorescence microscope (Nikon Inc, Melville, NY, USA) and warmed to 32°C over a 5-minute period in Tyrode’s solution. Fluorescence emission was captured using a high speed, digital QE CCD camera (TILL Photonics, Pleasanton, CA, USA). Each fiber was tested against a single dose of simvastatin, and peak fluorescence values were averaged across all fibers per group for each concentration.

### Simvastatin sensitivity and AICAR effectiveness in isolated fibers

To determine the effects of simvastatin dosing, YS and WT fibers loaded with Fura-2 AM were perfused for 2 minutes in warmed (32°C) Tyrode’s solution for recording of baseline Ca^2+^ levels, followed by a 3-minute exposure at specified doses of simvastatin. Separately, isolated fibers used to test AICAR’s effectiveness at preventing the statin-modulated change in Ca^2+^ were pre-incubated in 1 mM AICAR in conjunction with the 1-hour incubation in DMEM/Fura-2 AM before exposure to 500 μM and 1 mM simvastatin in the YS and WT, respectively. Fura-2 fluorescence was recorded and converted to cytosolic Ca^2+^ values as previously reported [16].

### 4-CMC-induced Ca^2+^ store depletion in isolated fibers

To evaluate the effects of simvastatin on SR Ca^2+^ store depletion, isolated fibers were exposed to 4-chloro-m-cresol (4-CmC) immediately after 3 minutes of incubation in 500 μM simvastatin. 4-CmC was applied to either YS or WT fibers at the dose found to induce maximal Ca^2+^ release without causing death of the individual fibers, which we determined to be 1 mM in the YS and 2.5 mM in the WT mice.

### Statistical analysis

A Student’s *t*-test was used for comparison between groups to test significance values of *P* <0.05 (*), *P* <0.01 (**), and *P* <0.001 (***). Dose–response curves were fit using 4-parameter (oxygen consumption (VO_2_)) or 3-parameter (single-fiber dose–response) Hill function curves in SigmaPlot, version 12.0 (Systat Software, San Jose, CA, USA). YS data was additionally fitted with a biphasic function using GraphPad Prism, version 6 (GraphPad Software, La Jolla, CA, USA).

## Results

### Simvastatin triggers HMR in YS mice

**Figure 1:**
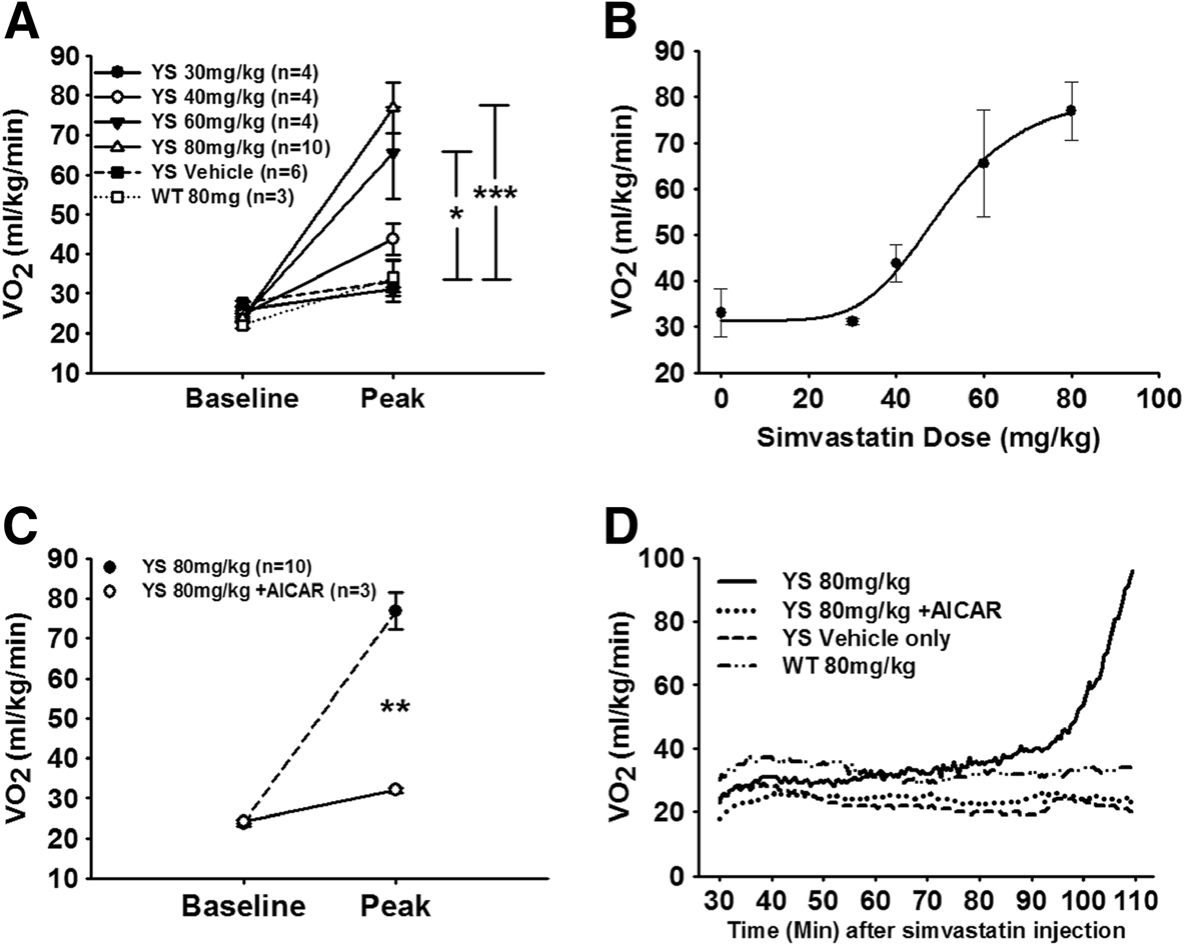
**An acute dose of simvastatin at 32°C results in higher peak VO**_**2 **_**levels in YS compared to WT mice. (A)** IP injection of simvastatin triggers significantly higher peak VO_2_ values at 60 mg/kg (*P <*0.05) and 80 mg/kg (*P* <0.001) compared to vehicle-only injection. **(B)** Curve-fit of increasing simvastatin doses in YS mice. **(C)** Pre-treatment with 600 mg/kg AICAR results in significantly (*P <*0.01) lower peak VO_2_ values when administered 20 minutes after simvastatin treatment in YS mice. **(D)** Representative VO_2_ tracings of YS mice receiving 80 mg/kg of simvastatin show increasingly higher VO_2_ values than YS mice treated with both 80 mg/kg simvastatin and 600 mg/kg AICAR, vehicle-only (DMSO), or WT mice treated with 80 mg/kg simvastatin. AICAR, 5-aminoimidazole-4-carboxamide ribonucleoside; DMSO, dimethyl sulfoxide; VO_2_, oxygen consumption; WT, wild type; YS, Y524S.

We previously demonstrated that changes in VO_2_ could be used to detect the HMR response in the YS mice exposed to elevated environmental temperatures [15]. This approach allows early detection of the HMR and allows the mice to be euthanized prior to a full body contraction and death. To determine if statins also trigger an HMR response, we injected mice with an acute dose of simvastatin (IP 30 to 80 mg/kg) and placed the mice in the chamber (32°C, a thermoneutral temperature that does not trigger HMR in the untreated YS mice) of the indirect calorimeter and measured VO_2_ as a function of time after injection. All YS mice injected with 60 or 80 mg/kg simvastatin exhibited subsequent signs of HMR, which included increased VO_2_ (Figure 1A), severe muscle contractures and increased heat production. After injection with simvastatin, a significantly higher peak VO_2_ occurred in YS mice receiving 60 mg/kg (*P* <0.05) and 80 mg/kg (*P* <0.001) doses when compared against YS mice injected with the vehicle. Figure 1B shows the dose–response curve for peak VO_2_ as a function of simvastatin dose in the YS mice.

To determine if the statin-induced HMR event was similar to heat-induced HMR in the YS mice, we injected the YS mice with 80 mg/kg of simvastatin followed by 600 mg/kg of AICAR, which we have previously shown to prevent temperature-induced HMR in the YS mice by decreasing Ca^2+^ leak from RyR1 [15]. AICAR eliminated the statin-associated HMR in YS mice by preventing the significant (*P* <0.01) increase in VO_2_ that occurs in YS mice not receiving the AICAR treatment (Figure 1C).

### Myofibrillar Ca^2+^ leak is more sensitive to simvastatin in YS compared to WT muscle fibers

**Figure 2:**
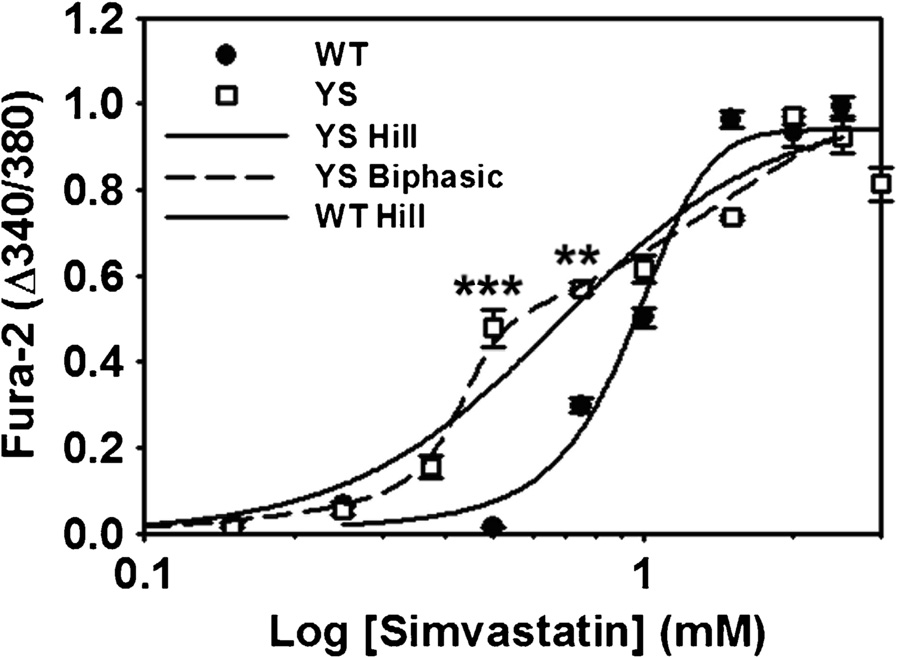
**Isolated fibers from YS mice exhibit increased sensitivity to simvastatin compared to WT mice.** Dose–response curves from isolated WT and YS FDB fibers incubated for 3 minutes in respective doses of simvastatin. Data points reflect peak cytosolic Ca^2+^ change from baseline, indicating that fibers from YS mice respond to simvastatin at lower doses than WT fibers. Fibers were used only at a single simvastatin concentration. Each data point represents the mean cytosolic Ca^2+^ response from a minimum of three fibers taken from three separate mice. Ca^2+^, calcium; FDB, flexor digitorum brevis; WT, wild type; YS, Y524S.

The strong protective effect of AICAR on the simvastatin response of the YS mice suggests that statin-induced HMR in these mice is likely due to altered Ca^2+^ handling within the myofiber. We tested the effects of simvastatin in isolated FDB fibers of YS and WT mice using the fluorescent dye Fura-2 to assess changes in cytosolic Ca^2+^ concentrations. We found that simvastatin triggered higher cytosolic Ca^2+^ levels in YS fibers at lower concentrations (500 μM (*P <*0.001) and 750 μM (*P <*0.01)) than in WT FDB fibers (Figure 2). As previously shown with human fibers [7], WT fibers displayed increased Ca^2+^ in response to higher doses of simvastatin (1.5 mM (*P* <0.01)). The concentration response curves in the YS and WT mice were best fit using a Hill function (3-parameter) with a resulting EC_50_ of 0.6 mM in the YS and 0.9 mM in the WT mice. Since the YS fibers are from heterozygous mice, the Ca^2+^ response reflects the heterogeneous response from a mixture of mutant channels (in various combinations of mutation and WT subunits) and WT channels. Using a 2-site model, we obtain EC_50_s of 0.4 and 0.9 mM. Ca^2+^ concentrations were calculated from the Fura-2 fluorescence as described in Methods.

### Simvastatin depletes SR Ca^2+^ stores in FDB fibers isolated from YS mice

**Figure 3:**
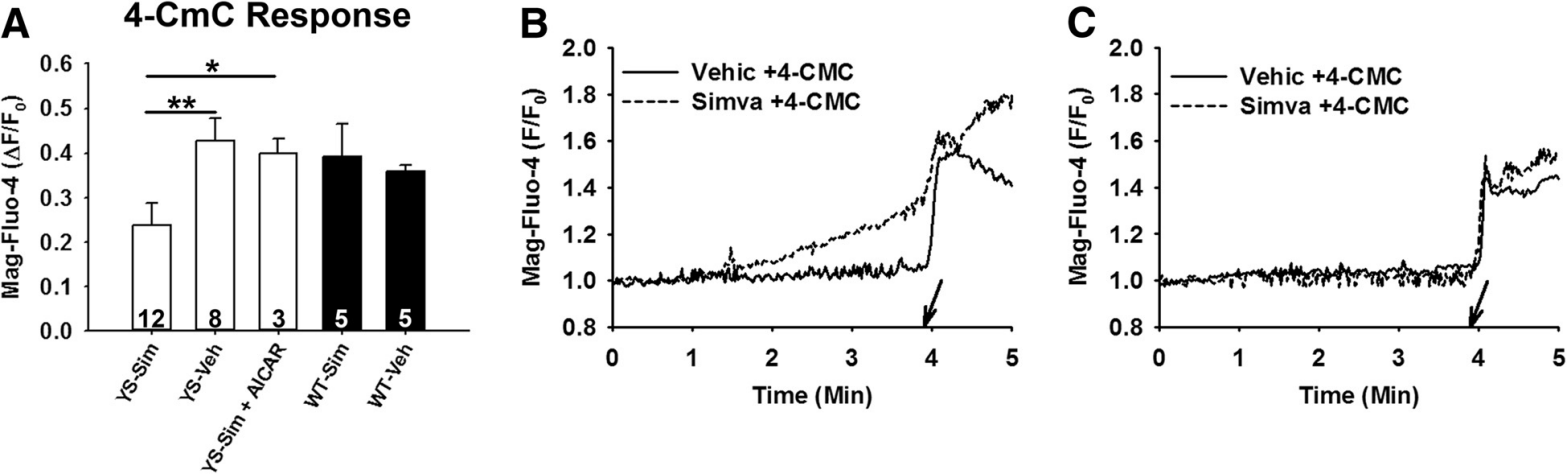
**Pre-incubation with simvastatin decreases the 4-CmC-modulated cytosolic Ca**^**2+**^**response in isolated FDB fibers from YS compared to WT mice.** Represented as the change (Δ) from baseline to peak values, **(A)** shows that upon exposure to 1 mM 4-CmC those YS fibers incubated for 3 minutes in 500 μM simvastatin (YS-Sim) release significantly less Ca^2+^ from the SR than YS fibers receiving vehicle-only (YS-Veh) incubation (*P* <0.01) and from YS fibers incubated in 1 mM AICAR followed by 500 μM simvastatin (YS-Sim + AICAR) (*P* <0.05). Numbers represent total fibers used per group from a minimum of three mice. **(B)** YS and **(C)** WT show representative Mag-fluo-4 fluorescence tracings in single fibers exposed to either simvastatin or vehicle. Arrows indicate the time point at which 4-CmC was applied to the fibers. AICAR, 5-aminoimidazole-4-carboxamide ribonucleoside; 4-CmC, 4-chloro-m-cresol; Ca^2+^, calcium; FDB, flexor digitorum brevis; SR, sarcoplasmic reticulum; WT, wild type; YS, Y524S.

Ca^2+^ stores in YS FDB fibers are decreased by exposure to elevated temperatures [17]. To determine if a reduction in Ca^2+^ stores occurs with simvastatin, we used Mag-fluo-4, a low-affinity Ca^2+^ indicator, and 4-CmC to assess the readily releasable SR Ca^2+^ stores [18]. 4-CmC was applied to isolated fibers immediately after a 3-minute incubation with simvastatin. We found a significant (*P* <0.05) decrease in the readily releasable Ca^2+^ stores in YS fibers exposed to 500 μM simvastatin compared with YS fibers exposed to vehicle-only (Figure 3), while no difference was found in WT fibers at this concentration of simvastatin. This finding suggests that the increased cytosolic Ca^2+^ levels in the YS mice that occur after exposure to simvastatin are due to SR Ca^2+^ leak leading to SR Ca^2+^ store depletion.

**Figure 4:**
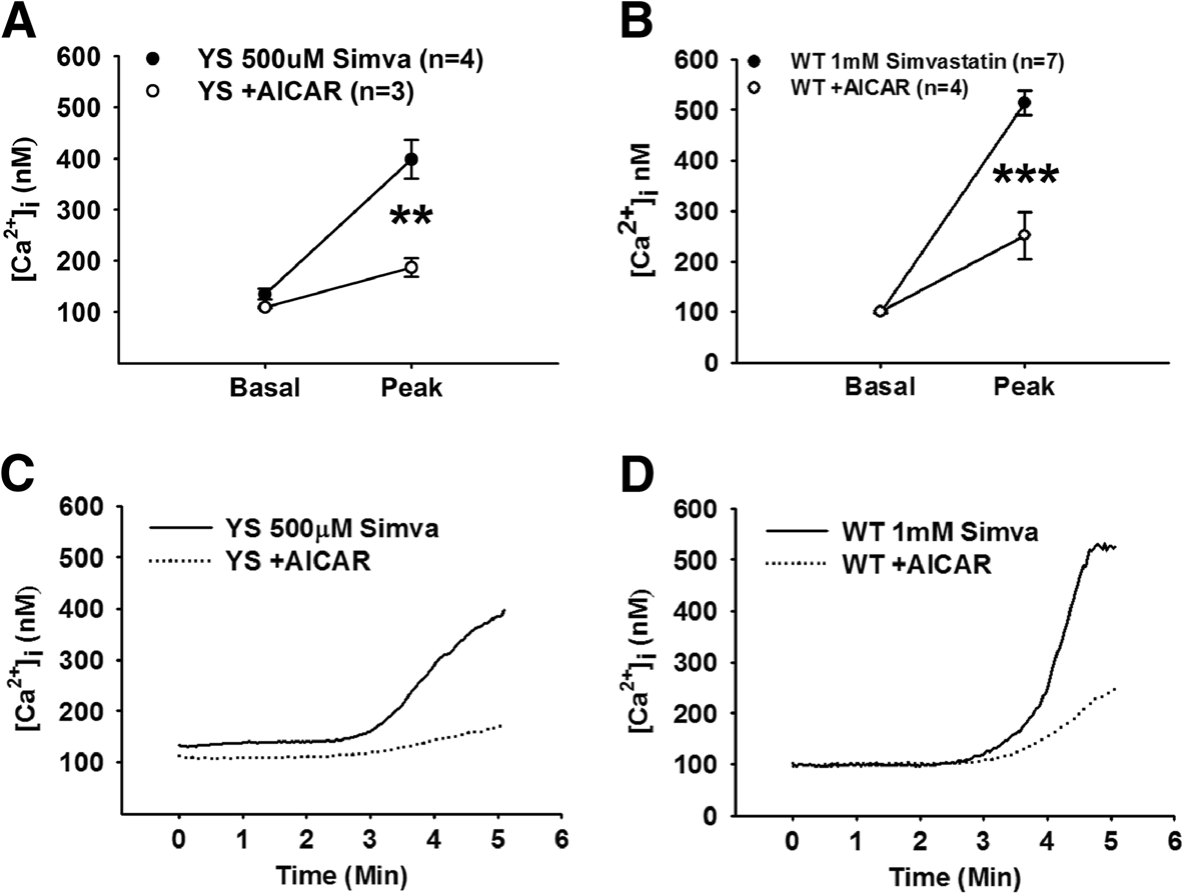
**Pre-treatment with AICAR reduces the cytosolic Ca**^**2+ **^**response to simvastatin.** Pre-incubation with AICAR prevents Ca^2+^ release in **(A)** YS fibers exposed to 500 μM simvastatin (*P* <0.01) and in **(B)** WT fibers exposed to 1 mM simvastatin (*P* <0.001). **(C)** YS and **(D)** WT show change in Ca^2+^ concentration for AICAR-treated (dashed) and untreated (solid) fibers after exposure to either 500 μM (YS) or 1 mM (WT) simvastatin at 2 minutes . AICAR, 5-aminoimidazole-4-carboxamide ribonucleoside; Ca^2+^, calcium; WT, wild type; YS, Y524S.

We assessed the ability of AICAR to regulate the simvastatin-induced increase in Ca^2+^ leak in the YS fibers. Isolated YS fibers were incubated with 1 mM AICAR prior to incubation with 500 μM simvastatin. As shown in Figure 4A, Ca^2+^ stores were protected from the simvastatin-induced depletion by prior administration of AICAR (*P* <0.01). We determined if AICAR could also prevent the simvastatin-induced Ca^2+^ release at higher simvastatin doses in WT mice (Figure 4B). When WT fibers were incubated with 1 mM simvastatin, we found that AICAR pre-treatment also greatly decreased Ca^2+^ release in WT fibers (*P <*0.001), suggesting that statins have the potential to trigger Ca^2+^ release in normal fibers but require higher simvastatin concentrations than YS fibers and that AICAR may be a useful intervention for SIM even in patients without RyR1 mutations.

## Discussion

Despite the prevalence of statin myopathies, a mechanism to explain the underlying trigger has remained elusive. The current study’s objective was to determine whether a MH-associated defect in RyR1 increased sensitivity to simvastatin and whether AICAR, which prevents heat-induced HMR in the YS mice, blocked the response to simvastatin. We show that the YS mice display an MH-like response (elevated VO_2_, sustained muscle contractures, elevated body temperature) to an acute dose of simvastatin, and the degree of response is dose-dependent. Simvastatin also enhances SR Ca^2+^ leak and SR Ca^2+^ store depletion in FDB fibers from both YS and WT mice but the response in WT mice requires higher concentrations of simvastatin. In FDB fibers from both YS and WT mice, the response to simvastatin was prevented by AICAR, suggesting that even in WT fibers the effect of simvastatin involves RyR1.

AICAR is a known activator of the energy sensing kinase, AMP-activated protein kinase (AMPK). We recently demonstrated, however, that AICAR also has a direct effect on RyR1 and rescues the YS mice from heat-induced sudden death independent of AMPK activation [15]. We now demonstrate that treatment of YS mice with AICAR, which decreases Ca^2+^ leak in the presence of cellular levels of ATP [15], prevents the simvastatin-associated increases in VO_2_ and heat production as well as greatly attenuates Ca^2+^ leak from the SR upon exposure of FDB fibers to simvastatin. AICAR also largely eliminates the statin-induced Ca^2+^ release in healthy WT mice. These results suggest that AICAR might also be a potential therapeutic intervention to prevent statin myopathies associated with RyR1 mutations in sensitive individuals and protect against myopathies arising from high statin doses in individuals without RyR1 mutations.

### YS mutation explains clinical symptoms of SIM

Alterations in Ca^2+^ signaling with simvastatin could explain many of the symptoms associated with SIM in humans including muscle fatigue, cramping, and increased levels of circulating CK. Depletion of stores contributes to fatigue, while increased resting Ca^2+^ is known to trigger Ca^2+^ release and muscle contraction, giving rise to muscle cramping similar to that which occurs in Brody disease. Brody disease results from a reduction in the number and activity of sarco/endoplasmic reticulum Ca^2+^-ATPase (SERCA) proteins in skeletal muscle, which inhibits the re-uptake of cytosolic Ca^2+^ during muscle activity [19,20]. Individuals afflicted with Brody disease complain of fatigue as well as muscle cramping that is exacerbated during periods of increased activity such as exercise [19]. These symptoms reflect those commonly reported among individuals experiencing SIM. Separately, elevated circulating CK levels among individuals experiencing SIM can also be explained by rhabdomyolysis triggered by the statin-modulated increase in cytosolic Ca^2+^ levels and activation of calpains [21,22]. Elevated CK levels are commonly experienced by individuals experiencing SIM.

### Clinical relevance

An acute dose of simvastatin increases cytosolic Ca^2+^ levels within the myofiber and this increase occurs at lower simvastatin concentrations in the presence of a RyR1 mutation associated with MH in humans. The prevalence of genetic abnormalities capable of causing MH has been estimated to be as low as 1:3,000 [12]. Whereas the incidence of SIM is relatively low (approximately 10%) among the millions of statin users, it is highly possible that those individuals exhibiting signs and symptoms of SIM are harboring an underlying RyR1 myopathy. Guis *et al*. showed that seven of nine individuals exhibiting symptoms of severe statin myopathy were found to have a positive IVCT, indicative of an underlying RyR1 abnormality [11]. Therefore, further research is needed to determine whether individuals experiencing SIM also have mutations in RyR1. If true, drugs such as AICAR that modulate RyR1 activity can be investigated as a potential therapy for these individuals, which may allow continued statin use without the side effects associated with SIM.

## Conclusions

The YS mutation in RyR1 increases the sensitivity to the cholesterol-lowering medication simvastatin. This sensitivity is marked by systemic increases in VO_2_, muscle contractures and heat production due to a temporal release of Ca^2+^ into the cytosol from the SR. Pharmaceutical interventions that decrease Ca^2+^ leak from RyR1 (such as AICAR) prevent both the systemic manifestation of SIM and the statin-induced Ca^2+^ release from the SR in single fibers. We show that RyR1 mutation increases sensitivity to SIM, suggesting that individuals affected by SIM could harbor underlying RyR1 mutations and that AICAR may be an effective therapeutic intervention.

## Abbreviations

4-CmC: 4-chloro-m-cresol; AICAR: 5-aminoimidazole-4-carboxamide ribonucleoside; AMPK: AMP-activated protein kinase; BTS: 4-methyl-N-(phenylmethyl)benzenesulfonamide; Ca2+: Calcium; CK: Creatine kinase; DMEM: Dulbecco’s modified Eagle’s medium; DMSO: Dimethyl sulfoxide; EC50: Half maximal effective concentration; EDTA: Ethylenediaminetetraacetic acid; EtOH: Ethanol; FDB: flexor digitorum brevis; Fura-2 AM: Fura-2 acetoxymethyl ester; HMG-CoA: 3-hydroxy-3-methylglutaryl coenzyme-A; HMR: Hypermetabolic response; IP: Intraperitoneal; IVCT: *In vitro* contracture test; LDL: Low-density lipoprotein; MH: Malignant hyperthermia; RyR1: Ryanodine receptor type 1; SERCA: Sarco/endoplasmic reticulum Ca^2+^−ATPase; SIM: Statin-induced myopathy; SR: Sarcoplasmic reticulum; VO2: Oxygen consumption; VO2 max: Maximum oxygen consumption; WT: Wild type; YS: Y524S.

## Competing interests

The authors declare that they have no competing interests.

## Authors’ contributions

MK conceived and developed the study, conducted indirect calorimetry experiments, assisted with Ca^2+^ imaging experiments, performed statistical analyses, and prepared the draft manuscript. ADA conducted Ca^2+^ imaging experiments, assisted with analyses, prepared data, and assisted with manuscript preparation. SLH created and maintained the YS mouse line, assisted with study design and data interpretation, and assisted with manuscript preparation. All authors read and approved the final manuscript.

## References

[B1] Joy T Hegele R Narrative review: statin-related myopathy Ann Intern Med 2009 150 12 858 868 10.7326/0003-4819-150-12-200906160-00009 19528564

[B2] Sathasivam S Lecky B Statin induced myopathy BMJ 2008 337 1159 1162 10.1136/bmj.a2286 18988647

[B3] Venero C Thompson P Managing statin myopathy Endocrinol Metab Clin North Am 2009 38 1 121 136 10.1016/j.ecl.2008.11.002 19217515

[B4] Ghatak A Faheem O Thompson PD The genetics of statin-induced myopathy Atherosclerosis 2010 210 2 337 343 10.1016/j.atherosclerosis.2009.11.033 20042189

[B5] Sirvent P Fabre O Bordenave S Hillaire-Buys D Raynaud De Mauverger E Lacampagne A Mercier J Muscle mitochondrial metabolism and calcium signaling impairment in patients treated with statins Toxicol Appl Pharmacol 2012 259 2 263 268 10.1016/j.taap.2012.01.008 22269104

[B6] Sirvent P Mercier J Lacampagne A New insights into mechanisms of statin-associated myotoxicity Curr Opin Pharmacol 2008 8 3 333 338 10.1016/j.coph.2007.12.010 18243052

[B7] Sirvent P Bordenave S Vermaelen M Roels B Vassort G Mercier J Raynaud E Lacampagne A Simvastatin induces impairment in skeletal muscle while heart is protected Biochem Biophys Res Commun 2005 338 3 1426 1434 10.1016/j.bbrc.2005.10.108 16271704

[B8] Sirvent P Mercier J Vassort G Lacampagne A Simvastatin triggers mitochondria-induced Ca2+ signaling alteration in skeletal muscle Biochem Biophys Res Commun 2005 329 3 1067 1075 10.1016/j.bbrc.2005.02.070 15752763

[B9] Inoue R Tanabe M Kono K Maruyama K Ikemoto T Endo M Ca2+-releasing effect of cerivastatin on the sarcoplasmic reticulum of mouse and rat skeletal muscle fibers J Pharmacol Sci 2003 93 3 279 288 10.1254/jphs.93.279 14646245

[B10] Metterlein T Schuster F Tadda L Hager M Roewer N Anetseder M Statins alter intracellular calcium homeostasis in malignant hyperthermia susceptible individuals Cardiovasc Ther 2010 28 6 356 360 10.1111/j.1755-5922.2010.00237.x 20955541

[B11] Guis S Figarella-Branger D Mattei JP Nicoli F Le Fur Y Kozak-Ribbens G Pellissier JF Cozzone PJ Amabile N Bendahan D In vivo and in vitro characterization of skeletal muscle metabolism in patients with statin-induced adverse effects Arthritis Rheum 2006 55 4 551 557 10.1002/art.22100 16874775

[B12] Rosenberg H Davis M James D Pollock N Stowell K Malignant hyperthermia Orphanet J Rare Dis 2007 2 21 10.1186/1750-1172-2-21 17456235 PMC1867813

[B13] Chelu M Goonasekera S Durham W Tang W Lueck J Riehl J Pessah I Zhang P Bhattacharjee M Dirksen R Hamilton SL Heat- and anesthesia-induced malignant hyperthermia in an RyR1 knock-in mouse FASEB J 2006 20 329 330 16284304 10.1096/fj.05-4497fje

[B14] Leung B Sattar N Crilly A Prach M McCarey D Payne H Madhok R Campbell C Gracie J Liew F McInnes I A novel anti-inflammatory role for simvastatin in inflammatory arthritis J Immunol 2003 170 1524 1530 12538717 10.4049/jimmunol.170.3.1524

[B15] Lanner JT Georgiou DK Dagnino-Acosta A Ainbinder A Cheng Q Joshi AD Chen Z Yarotskyy V Oakes JM Lee CS Monroe TO Santillan A Dong K Goodyear L Ismailov II Rodney GG Dirksen RT Hamilton SL AICAR prevents heat-induced sudden death in RyR1 mutant mice independent of AMPK activation Nat Med 2012 18 2 244 251 10.1038/nm.2598 22231556 PMC3274651

[B16] Dagnino-Acosta A Guerrero-Hernández A Variable luminal sarcoplasmic reticulum Ca^2+^buffer capacity in smooth muscle cells Cell Calcium 2009 46 188 196 10.1016/j.ceca.2009.07.005 19679350

[B17] Durham W Aracena-Parks P Long C Rossi A Goonasekera S Boncompagni S Galvan D Gilman C Baker M Shirokova N Protasi F Dirksen R Hamilton S RyR1 S-nitrosylation underlies environmental heat stroke and sudden death in Y522S RyR1 knockin mice Cell 2008 133 53 65 10.1016/j.cell.2008.02.042 18394989 PMC2366094

[B18] Herrmann-Frank A Richter M Lehmann-Horn F 4-Chloro-m-cresol: a specific tool to distinguish between malignant hyperthermia-susceptible and normal muscle Biochem Pharmacol 1996 52 149 155 10.1016/0006-2952(96)00175-X 8678899

[B19] Vattemi G Gualandi F Oosterhof A Marini M Tonin P Rimessi P Neri M Guglielmi V Russignan A Poli C van Kuppevelt T Ferlini A Tomelleri G Brody disease: insights into biochemical features of SERCA1 and identification of a novel mutation J Neuropathol Exp Neurol 2010 69 3 246 252 10.1097/NEN.0b013e3181d0f7d5 20142766

[B20] Voermans N Laan A Oosterhof A van Kuppevelt T Drost G Lammens M Kamsteeg E Scotton C Gualandi F Guglielmi V van den Heuvel L Vattemi G van Engelen B Brody syndrome: a clinically heterogeneous entity distinct from Brody disease: a review of literature and a cross-sectional clinical study in 17 patients Neuromuscul Disord 2012 22 944 954 10.1016/j.nmd.2012.03.012 22704959

[B21] Belcastro A Shewchuk L Raj D Exercise-induced muscle injury: a calpain hypothesis Mol Cell Biochem 1998 179 1–2 135 145 9543356 10.1023/a:1006816123601

[B22] Raastad T Owe S Paulsen G Enns D Overgaard K Crameri R Kiil S Belcastro A Bergersen L Hallén J Changes in calpain activity, muscle structure, and function after eccentric exercise Med Sci Sports Exerc 2010 42 1 86 95 10.1249/MSS.0b013e3181ac7afa 20010126

